# RETRACTED: Ardissino et al. Short-Term Effect of Nutraceutical Fruit Juices on Lipid Metabolism in Patients with Acquired Hypercholesterolemia. *Int. J. Mol. Sci*. 2023, *24*, 7358

**DOI:** 10.3390/ijms27209087

**Published:** 2026-10-14

**Authors:** Diego Ardissino, Alessandro Colletti, Marzia Pellizzato, Gianna Pagliari, Francesco Di Pierro, Giancarlo Cravotto

**Affiliations:** 1 Cardiothoracic and Vascular Department, University Hospital of Parma, 43126 Parma, Italy; diego.ardissino@unipr.it; 2 Department of Drug Science and Technology, University of Turin, 10125 Turin, Italy; alessandro.colletti@unito.it; 3 Italian Society of Nutraceutical Formulators (SIFNut), 31033 Treviso, Italy; marzia.pellizzato@gmail.com; 4 Galfrè Giacomo S.A.S., 12032 Barge, Italy; giannapagliari36@gmail.com; 5 Scientific & Research Department, Velleja Research, 20125 Milan, Italy; f.dipierro@vellejaresearch.com

The journal retracts the article titled “Short-Term Effect of Nutraceutical Fruit Juices on Lipid Metabolism in Patients with Acquired Hypercholesterolemia” [1], cited above.

Following publication, concerns were brought to the attention of the Editorial Office regarding the ethical oversight of the study and the completeness of the potential competing interest disclosures.

In accordance with the journal’s standard procedures, the Editorial Office and Editorial Board conducted an investigation, which identified inconsistencies between the description of the study and its reported design. A further assessment by the Editorial Board found no documented evidence that appropriate ethical review had been obtained prior to the commencement of the study. Although the authors cooperated with the investigation and provided supporting materials, the Editorial Board determined that the explanations and documentation provided did not sufficiently address the concerns identified. As a result, the study was found to be non-compliant with MDPI’s policies on research involving human subjects. Therefore, the Editorial Board has decided to retract this article in accordance with MDPI’s retraction policy.

This retraction was approved by the Editor-in-Chief of the *International Journal of Molecular Sciences*.

The authors have been informed of this retraction and have indicated their disagreement with this decision.
